# A simultaneous electroencephalography and eye-tracking dataset in elite athletes during alertness and concentration tasks

**DOI:** 10.1038/s41597-022-01575-0

**Published:** 2022-08-02

**Authors:** Xinzhen Pei, Guiying Xu, Yunhui Zhou, Luna Tao, Xiaozhu Cui, Zhenyu Wang, Bingru Xu, An-Li Wang, Xi Zhao, Haijun Dong, Yan An, Yang Cao, Ruxue Li, Honglin Hu, Yuguo Yu

**Affiliations:** 1 Human Phenome Institute, State Key Laboratory of Medical Neurobiology and Ministry of Education Frontiers Center for Brain Science, School of Life Science, Research Institute of Intelligent Complex Systems, and Institute of Science and Technology for Brain-Inspired Intelligence, Fudan University, Shanghai, China; 2 Shanghai Advanced Research Institute, Chinese Academy of Sciences, Shanghai, China; 3 University of Chinese Academy of Sciences, Beijing, China; 4 Shanghai Competitive Sports Training Management Center, Shanghai, China; 5 Shanghai Research Institute of Sports Science (Shanghai Anti-doping Agency), Shanghai, China; 6 Department of Psychiatry, Icahn School of Medicine at Mount Sinai, New York, NY USA; 7 Shanghai Normal University, Shanghai, China

**Keywords:** Human behaviour, Signal processing, Human behaviour

## Abstract

The dataset of simultaneous 64-channel electroencephalography (EEG) and high-speed eye-tracking (ET) recordings was collected from 31 professional athletes and 43 college students during alertness behavior task (ABT) and concentration cognitive task (CCT). The CCT experiment lasting 1–2 hours included five sessions for groups of the Shooting, Archery and Modern Pentathlon elite athletes and the controls. Concentration targets included shooting target and combination target with or without 24 different directions of visual distractors and 2 types of music distractors. Meditation and Schulte Grid trainings were done as interventions. Analysis of the dataset aimed to extract effective biological markers of eye movement and EEG that can assess the concentration level of talented athletes compared with same-aged controls. Moreover, this dataset is useful for the research of related visual brain-computer interfaces.

## Abstract (summary)

**Table:** 

Measurement(s)	brain activity and eye movements measurement
Technology Type(s)	electroencephalography and eye-tracking
Factor Type(s)	electroencephalography (EEG) • eye-tracking (ET)
Sample Characteristic - Organism	Human
Sample Characteristic - Location	China

## Background & Summary

Attention is complex and has several different forms including focused, sustained, selective, alternating and divided attentions^1^. In sport psychology, the ability to focus on the task at hand while ignoring distractions from both external and internal sources is vital for successful athletic performance^2^. Cox remarked that few topics in sport psychology were as important as understanding “attention or concentration” in athletes^3^. Moran *et al*., suggested that attentional factors as “sustained alertness and freedom from distraction” were vital ingredients of athletic performance. Hosseiny *et al*., expressed the opinion that one of the sports which was highly depended on the attentional focus was shooting^4^. Archery, like shooting, was also regarded as closed skills and precision sports^5^. Modern pentathlon included pistol shooting, but it was a comprehensive sport and comprised other four events (fencing, swimming, horseback riding and running)^6^. Athletes of these three sports may have various requirement for concentration, especially the elite athletes. Previous studies also found that the professional shooters had more stable fixation than non-professional controls^7^. Accuracy of shooting performance (i.e. shooting position was as close as to the center of the target), in the case of shooting^8^ and archery^9^, was observed to be associated with alpha band oscillation in left cerebral hemisphere. The ability to adapt and refocus in the face of distractions was suggested to be one of key mental skills for distinguishing successful from unsuccessful athletes^10^. Given the importance of concentration in sports, many psychologists suggested that concentration skills may be enhanced through appropriate training, such as meditation^11,12^ and Schulte Grid Training^13,14^. However, the relative brain and behavior signatures of this potentially enhanced ability is not well studied. There are several brain imaging datasets of college athletes^15^, amateur chess^16^ or soccer players^17^ available publicly, but the datasets on concentration cognitive task and their brain activity datasets for professional athletes, especially national master level athletes of Shooting, Archery and Modern Pentathlon are lacking.

Attention can modulate the level of brain excitability. Evidence from a previous study showed that there was a nonlinear relationship between brain excitability and task performance across multiple species^18^. The performance may be linked to the optimal level of excitability, while either under- or over-excitation may degrade the performance^18,19^. Behaviorally, attention was also correlated with eye movements. For example, the amplitude of microsaccades decreased in long-term fixation training^20^ and showed an inverse correlation with the level of human concentration^21^. Good emotional state and sleep quality were conductive to the cognitive performance of athletes^22^. Previous studies observed that sleep disorder had a significantly negative association with attention^23^, and the resultant anxiety level was inversely correlated with athletic performance^24^. On the contrary, positive emotions were observed to be associated with good athletic performance, such as high energy, low fatigue, tension, depression, anger and confusion^25,26^.

In this study we simultaneously recorded 64-channel electroencephalograph (EEG) and high-speed (2000Hz) eye tracking (ET) data while participants were performing different visual focused tasks. Thirty-one elite athletes (shooting, archery and modern pentathlon) and 43 age-matched college students were recruited. Three questionnaires reflecting emotional, sleeping and anxious states were filled out by each participant before all the experiments. By deeply analyzing these EEG and ET data, we aimed to identify a set of key factors that tightly correlate to the concentration level of elite athletes as well as the controls group.

Moreover, research in noninvasive brain-computer interfaces (BCIs) presents considerable potential to provide a convenient and safe method to measure attention level. Significant progress in EEG BCI for attention level measurement has been made, for example EEG can be utilized to measure attentional demands^27^, accurate classifiers have been constructed to decode P300 event-related potentials^28^, driver’s focus of attention can be tracked through EEG during distracted driving^29^ etc. Nevertheless, the absence of a comprehensive public EEG BCI dataset designed for attention or concentration level detection has become a hindrance for further exploration of more intrinsic correlations between EEG and attention or concentration level. Thus, these simultaneous EEG and ET recordings with multiple tasks of elite athletes are valuable for further study of BCI algorithms.

## Methods

### Participants

Seventy-four participants were recruited in total. Thirty-one were athletes, including sixteen elite Shooting and Archery athletes (21.14 ± 2.63 years old, 7 female), fifteen Modern Pentathlon athletes (20.49 ± 3.79 years old, 6 female) from Shanghai Competitive Sports Training Management Center were recruited (Table 1). Forty-three college students (21.38 ± 1.8 years old, 19 female) with no history of professional sports training were recruited as controls. There were no significant differences in ages and gender between two groups (p > 0.05). The study protocol was approved by the Ethics Committee of the School of Life Sciences, Fudan University.

**Table 1:** Description of all the athletes in the dataset.

Participant No.	Age (years)	Gender	Sport/Event	Professional Level
Sub301	24.5	Female	Archery	National 1^st^ Level
Sub302	19.9	Male	Archery	National Master
Sub303	21.1	Female	Archery	National 1^st^ Level
Sub304	19.6	Female	Archery	National Master
Sub305	21.5	Male	Shooting/Pistol	National 1^st^ Level
Sub307	24.4	Male	Shooting/Pistol	National 1^st^ Level
Sub308	18.3	Male	Archery	National Master
Sub309	21	Male	Archery	National 1^st^ Level
Sub311	24.4	Male	Shooting/Pistol	National Master
Sub313	23.6	Male	Shooting/Pistol	National 1^st^ Level
Sub314	18.1	Male	Shooting/Pistol	National 1^st^ Level
Sub315	21.2	Male	Shooting/Rifle	National 1^st^ Level
Sub317	17.4	Female	Shooting/Rifle	National 1^st^ Level
Sub318	18.2	Female	Shooting/Rifle	National 1^st^ Level
Sub319	25.4	Female	Shooting/Rifle	National Master
Sub321	19.7	Female	Shooting/Rifle	National 1^st^ Level
Sub502	23.9	Male	Modern Pentathlon	National Master
Sub504	24.4	Male	Modern Pentathlon	International Master
Sub505	27.8	Male	Modern Pentathlon	International Master
Sub506	26.4	Male	Modern Pentathlon	National Master
Sub507	16.5	Female	Modern Pentathlon	National 1^st^ Level
Sub508	19.2	Male	Modern Pentathlon	National 1^st^ Level
Sub510	19.6	Female	Modern Pentathlon	National Master
Sub511	16.3	Female	Modern Pentathlon	National 1^st^ Level
Sub512	17.4	Male	Modern Pentathlon	National 1^st^ Level
Sub513	15.2	Male	Modern Pentathlon	National 2^nd^ Level
Sub514	21.7	Male	Modern Pentathlon	National Master
Sub515	21	Male	Modern Pentathlon	National Master
Sub516	18.9	Female	Modern Pentathlon	National Master
Sub517	17.8	Female	Modern Pentathlon	National 2^nd^ Level
Sub518	21.2	Female	Modern Pentathlon	National Master

Note: International Master > National Master > National 1^st^ Level > National 2^nd^ Level.

All participants were informed about the purpose and the procedure of the study and signed informed consent. They were all aware that their data might make public without containing their personal information. Participants were all self-reported right-handed verified by the observations of using their hands in writing and performing tasks. None of them reported any history of mental illness. All data was anonymized and identified only by participants’ ID such as “Sub001”.

Prior to the experiments, all participants were asked to fill the demographic information and three questionnaires including Pittsburgh Sleep Quality Index (PSQI)^30^, Profile of Mood States (POMS)^31,32^ and the Competitive State Anxiety Inventory-2 (CSAI-2)^33^. Athletes additional provided the history of sports training and injuries. Since the participants in the control group were all college students, “the pre-competition state” for elites was changed to “pre-exam state” for the control group in the CSAI-2 questionnaire. For example, the question "I'm worried about the competition" for elites was changed to “I’m worried about the exam” for college students. The demographic information and results of questionnaires were stored in the excel format files named ‘Participants_Demographic_Information’, ‘Participants_CSAI-2_Results’, ‘Participants_POMS_Results’ and ‘Participants_PSQI_Results’, respectively (Fig. 4).

### Experimental procedures

During the experiment, participants were seated comfortably in an armchair in a soundproof room and weared a 64-channel EEG cap (Neuroscan, Australia) on their head. Stimuli were presented on a 32-inch (length: 0.697344 m; width: 0.392256 m) PHILIPS 325M1 LCD monitor at a resolution of 2560 × 1440 and a refresh rate of 165 Hz. Participants’ heads were fixed by a chin rest and were approximately 0.8 m in front of the screen. Stimuli were generated and presented using MATLAB (The Math Works, 1993) and the Psychophysics Toolbox^34^ running on a Window 7 (Microsoft) machine. Eye movements were recorded by an EyeLink Portable DUO eye-tracker (SR Research, Canada). Viewing was always binocular, 12 participants had only left eye recordings, 3 participants had only right eye recordings, and the rest 59 had binocular recordings. The sampling rate was always 2000Hz regardless of the number of eyes recorded. At the beginning of alertness or concentration tasks, each participant was required to perform a 13-point calibration routine until the average test-retest measurement error of single or both eyes fell below 0.5°.

**Fig. 1:**
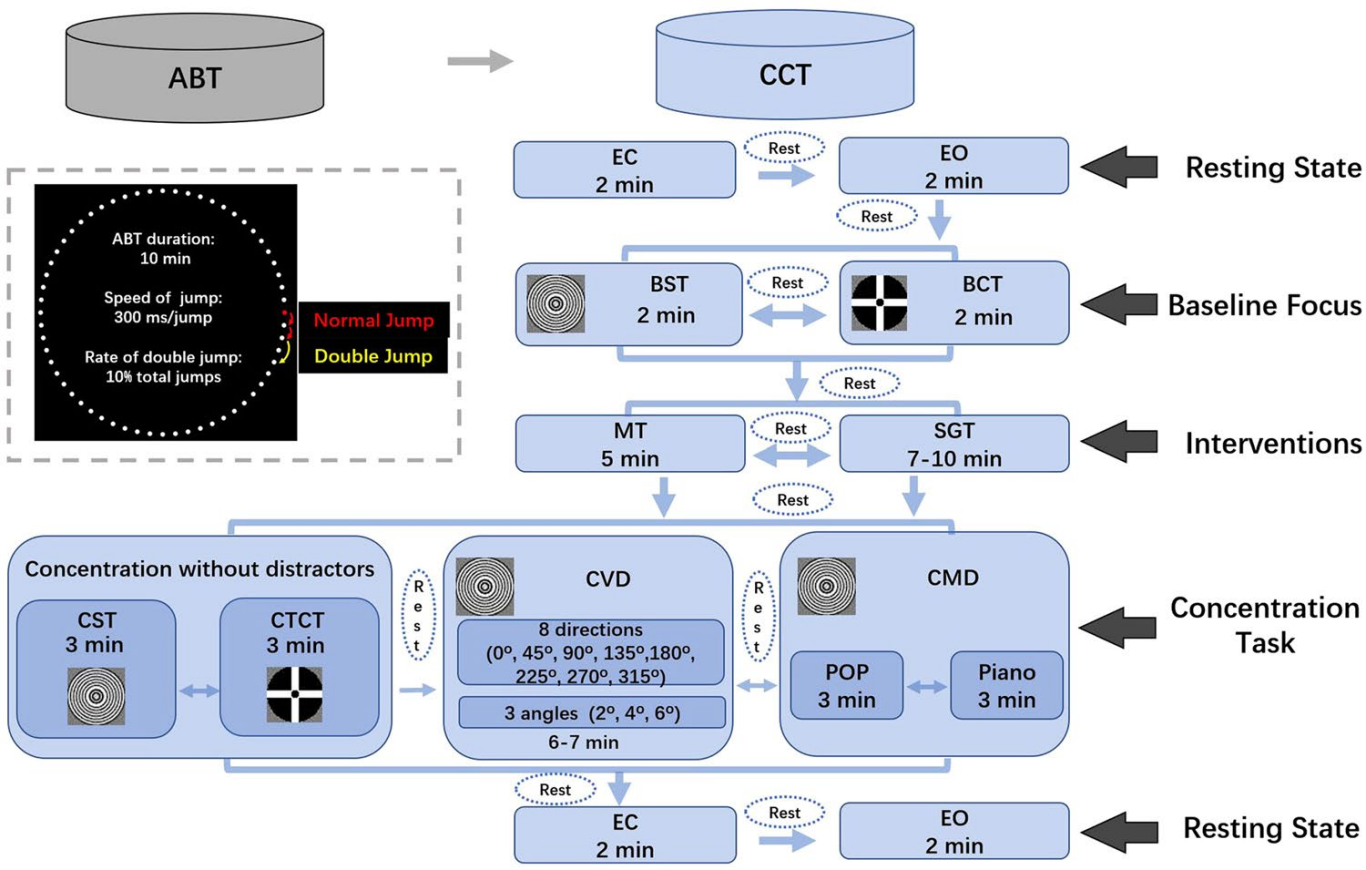
Schematic representation of experimental procedures and the data acquisition. (Abbreviations: ABT = Alertness Behavior Task; CCT = Concentration Cognitive Task; EC = Eye Closed; EO = Eye Open; BST = Baseline Shooting Target fixation; BCT = Baseline Combination Target fixation; MT = Meditation Training; SGT = Schulte Grid Training; CST = Concentration Shooting Target; CTCT = Combination Target Concentration Task; CVD = Concentration with Visual Distractors; CMD = Concentration with Music Distractors).

In the datasets there were two independent experiments which included tasks classified into different sessions. During each experiment, the simultaneous EEG and ET data of the tasks were continuously recorded and marked by triggers. The experimental procedures were illustrated in Fig. 1. The two experiments were a 10-minute Alertness Behavior Task (ABT) and a Concentration Cognitive Task (CCT, 70–90 minutes). The ABT experiment was a concentration behavior measurement which adapted from the Mackworth Clock Test^35^. The higher the response accuracy, the higher the level of concentration. The CCT experiment was designed according to the feature that shooters must try their best to focus the attention on shooting target center during the shooting process. The closer the eyes gaze to the center of the target and the longer the gaze time, the higher the concentration of the brain is considered to be. Given that the actual shooting competition field has some background noise interference, we added visual and auditory distractors to the CCT experiment in order to study the level of concentration under various conditions. In addition, we also aimed to find the effective methods to improve the level of concentration for athletes and coaches. Therefore, we have examined the post-training effects of some intervention methods, such as focusing on the best fixation target^36^, meditation training^11,12^ and Schulte Grid Training^13,14^ etc. based on published literatures. The set of total duration of both experiments was based on the real situations in shooting competitions (e.g., sixty match shots are fired within 75 minutes in the qualification of individual 10-m air pistol competitions set by International Shooting Sport Federation).

Before the experiments, the major experiment instructor illustrated clearly all the procedures (Fig. 1) to each participant and explicitly explained how to perform each task correctly in line with the following descriptions of the tasks. Meanwhile, on the screen there was one picture with a clear and standardized instruction of each task to make every participant to understand how to perform the tasks correctly. When the experiments started, the same instruction picture would be shown again at the beginning of each task. The instruction pictures were the same for all the participants and our instructor explained in language in full accordance with the pictures. Participants were allowed to take a self-paced break at every inter-task interval. A one dot drift-check procedure was run at the beginning of each task, and the eye-tracker was recalibrated when necessary. Detailed descriptions of each experiment are given below.

### Experiment 1: Alertness behavior task (ABT)

The ABT experiment, as a concentration behavior measurement, was adapted from the Mackworth Clock Test^35^. Sixty white dots with a diameter of 20 pixels were evenly distributed on an invisible circle with radius of 500 pixels on a black background. During the experiment, each white dot turned red for 300 ms and turned white for 300 ms in clockwise order. The leftmost dot turned red first. Normally the dots turned red consecutively and participants should not make any response. However, in randomly selected 10% of the cases, the red dot made a double jump (e.g., one white dot was skipped and did not turn red), and participants were required to respond as fast as possible by pressing the space key. The response time window was 1 second. We recorded the timestamp of all response events and double jump events during the experiment, and we paired them after the experiment finished. If a response event was found within 1 second after a double jump event, it was a hit. If multiple response events were found within 1 second after a single double jump event, the first response was a hit and the rest were false alarms. If there was only a single response event within 1 second after multiple double jump events, the response was a hit and the rest double jumps were misses. Misses and false alarms were classified as error. Participants were instructed to focus on the movement of the red dot only throughout the task. The duration of the ABT was 10 minutes.

**Fig. 2:**
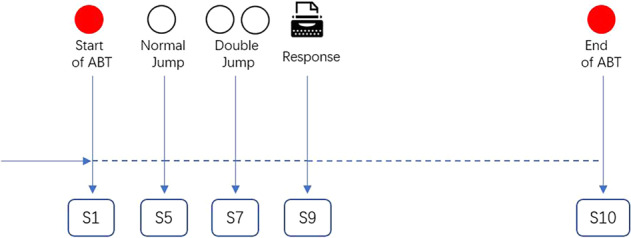
Triggers in EEG and ET data of the alertness behavior task (ABT).

We recorded the total number of double jumps, the number of misses and false responses for each participant. The triggers in EEG and ET data of ABT were shown in Fig. 2. Results of ABT were stored in the excel format file named ‘Participants_ABT_BehaviorResults’ (Fig. 4).

### Experiment 2: Concentration cognitive task (CCT)

The CCT experiment included 5 sessions. The first and fifth sessions were two 4-minute resting state sessions, each comprised a 2-minute eye-closed (EC) and a 2-minute eye-open (EO) task. The second baseline focus session contained a 2-minute Baseline Shooting Target (BST) fixation task and a 2-minute Baseline Cross-circle combination Target (BCT) fixation task^36^. The order of these two fixation tasks was randomized. The third interventions session contained a 5-minute Meditation Training (MT) task and a 7–10 minutes Schulte Grid Training (SGT) task which aimed to improve the participant’s ability to concentrate. The order of the interventions was randomized. The fourth concentration task session contained three tasks: Concentration without Distractors (CWD), Concentration with Visual Distractors (CVD) or Concentration with Music Distractors (CMD). In the tasks of no distractors, there were a 3-minute Concentration Shooting Target (CST) cognitive task and a 3-minute Combination Target Concentration Task (CTCT). The order of CVD and CMD tasks was randomized. In the CMD task, either pop or piano music was played as background auditory distractors. Participants were instructed to focus on the shooting target when performing both CVD and CMD tasks.

The triggers in EEG and ET data of each session were shown in Table 2. During the experiment, the background of the LCD screen was set to grey, the contrast was set to 50%, and the brightness was set to 0. Detailed descriptions of each task are given below.

**Table 2:** Descriptions of triggers used in the CCT experiment.

Session	Task			Start Trigger	End Trigger
Resting States	Eye Closed (EC)			S1	/
	Eye Open (EO)			S2	/
Baseline Focus	Shooting Target Fixation task (BST)			S10	S12
	Combination Target Fixation task (BCT)			S15	S17
Interventions	Meditation Training Task (MT)			S20	S22
	Schulte Grid Training Task (SGT)			S25	S27
Concentration Task	Concentration without Distractors	Shooting Target Concentration task (CST)		S30	S32
		Combination Target Concentration task (CTCT)		S35	S37
	Concentration with Visual Distractors (CVD)	Location (Radius Degree)	Location (Direction Degree)	/	/
		2	0	S41	S101
		4	0	S42	S102
		6	0	S43	S103
		2	45	S44	S104
		4	45	S45	S105
		6	45	S46	S106
		2	90	S47	S107
		4	90	S48	S108
		6	90	S49	S109
		2	135	S50	S110
		4	135	S51	S111
		6	135	S52	S112
		2	180	S53	S113
		4	180	S54	S114
		6	180	S55	S115
		2	225	S56	S116
		4	225	S57	S117
		6	225	S58	S118
		2	270	S59	S119
		4	270	S60	S120
		6	270	S61	S121
		2	315	S62	S122
		4	315	S63	S123
		6	315	S64	S124
	Concentration with Music Distractors (CMD)	POP Music Distractors (CMD_POP_)		S211	S212
		Piano Music Distractors (CMD_Piano_)		S221	S222
Resting States	Eye Closed (EC)			S1	/
	Eye Open (EO)			S2	/

### Session 1: Resting states

The CCT experiment started with two resting state tasks. They contained a 2-minute EC period and a 2-minute EO period. There was only one start trigger in each resting state task. The EC and EO states at the end were calculated offline using the 2 minutes interval. Participants were told to close their eye (EC period) or fixate at the “+” shown on the center of the screen (EO period) without making any additional thoughts. There was a short break between the two states. Because the triggers of the first and last sessions were the same, we distinguished them by the order of their appearance. The first “S1-S2” trigger pair marks the first resting state session.

### Session 2: Baseline focus

There were two fixation tasks (BST and BCT) in this session. The order of these two tasks was randomized.

### Task 2_1: Baseline shooting target fixation (BST)

Based on the size of the shooting target of the 10-meter air pistol in Olympics (seen STable 1 in the supplementary) and the distance between the participant and the screen (0.8 m), we rescaled the shooting target proportionately in relation to the center of the screen (Fig. 1). Participants were instructed to try their best to focus their attention on the second inner circle of 10 rings for 2 minutes. The closer the eyes gaze to the center of the target and the longer the gaze time, the higher the concentration of the brain is considered to be. Hence by calculating the fixation distance between the eye’s fixation location and the center of the shooting target, the level of concentration is estimated. This assessing method can be used in all the following target fixation tasks.

### Task 2_2: Baseline combination target fixation (BCT)

Previous studies found that a particular shape of a fixation target, such as a combination of a bull’s eye and cross hair, can minimize involuntary eye movements during fixation and facilitate stable fixation^36^. In our experiment, the diameter of the bull’s eye of the combination target was set to the same size as the 10 rings of the shooting target. The width of the cross hair was the diameter of 1 ring of the shooting target (Fig. 1). Participants were required to fixate on the bull’s eye for 2 minutes during the BCT task.

### Session 3: Interventions

There were two interventions in this session. The order of the interventions was randomized across participants.

### Task 3_1: Meditation training (MT)

When the participants were ready and pressed any key of the keyboard, they were instructed to close their eyes to prepare for meditation. About 1 second later, a beep sound was played from two speakers near the screen and prompted the participants to start meditation. They were requested to imagine that there was a shooting target in the middle of their eyebrows and imagine that they tried to focus their attention on the center of the imaginary shooting target, i.e., 10-ring. Before the MT intervention, participants were reminded that the EEG signals would be different if they didn’t meditate according to the instructions to guarantee MT quality as much as possible. The MT intervention lasted for 5 minutes.

### Task 3_2: Schulte grid training (SGT)

**Fig. 3:**
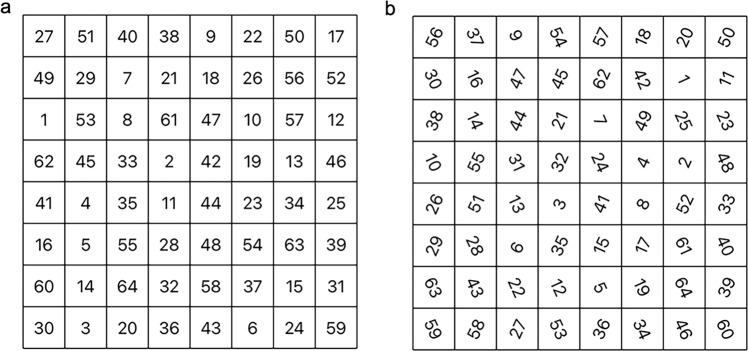
Schulte grid tests (8 × 8). (**a**) Forward direction type. (**b**) Rotation type.

**Fig. 4:**
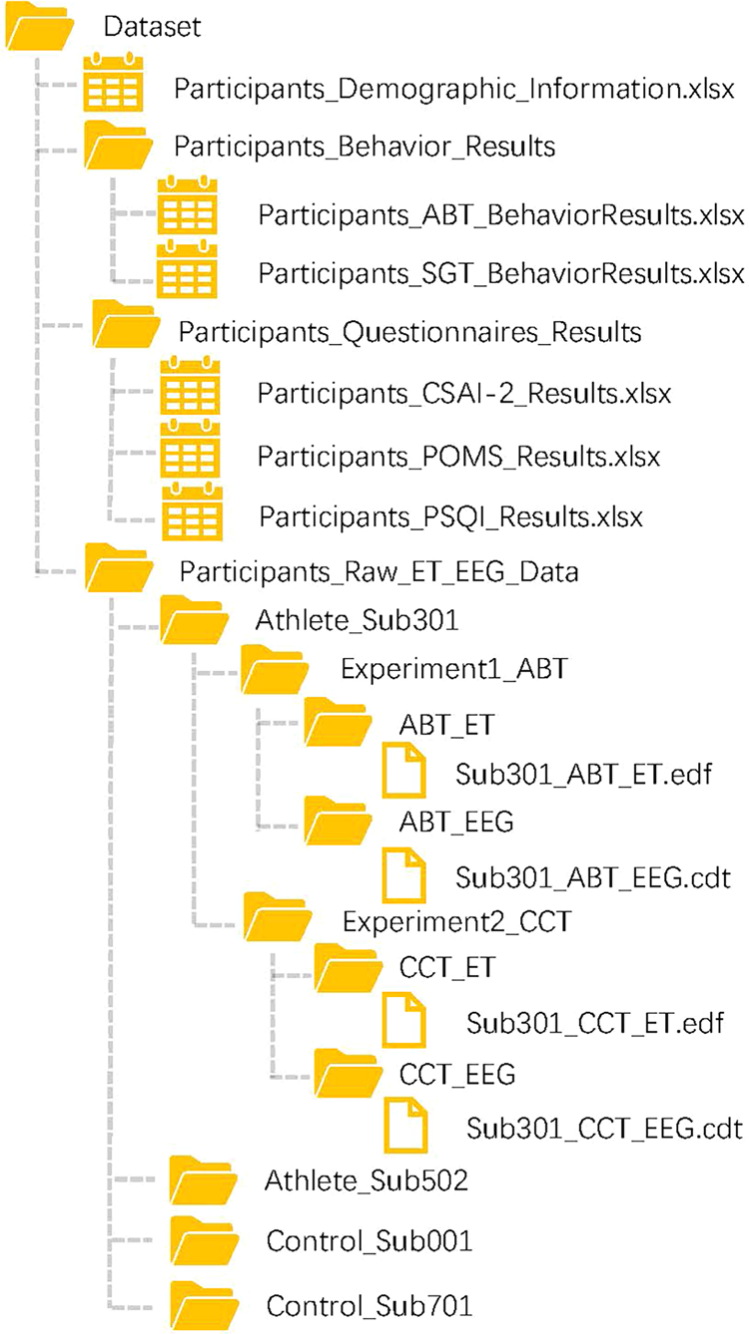
Directory structure of the dataset.

The Schulte Grid test is an effective science-based attention training method^13^. We selected two types of Schulte grid tests as the other intervention: 8 × 8 forward direction (Fig. 3a) and 8 × 8 rotation (Fig. 3b). Both Schulte Grid tests were performed in 64 squares on a 10.2 inches iPad (APPLE A2270, USA) with touch screen. Arabic numerals 1 to 64 were filled in them randomly. The differences between the two types of tests were the orientation of the numbers and their motions. One was forward and static, the other type was rotated in varied directions and moving. In both tests, participants should first find the right number from 1 to 64 in ascending order. That is, they had to first find where is the number “1”, then pressed the number “1” on the touch screen with their fingers. After they pressed the right number “1”, the tip text over the squares on the top of the iPad screen would show the next number “2” to let participants know which number they should find and press next. The task would be ended until they found the last number “64” and pressed correctly. The number didn’t disappear or being marked after pressed. Reaction times were recorded. The shorter reaction time indicated better attention. Results of SGT were stored in the excel spreadsheet named ‘Participants_SGT_BehaviorResults’ (Fig. 4).

### Session 4: Concentration task

This session was completed twice by all participants due to being performed once after each intervention task. Three tasks were included in this part. The session started with the concentration task without distractor, and followed by two concentration tasks with either visual or auditory distractors.

### Task 4_1: Concentration without distractors (CWD)

In this task, two types of targets, a shooting target and a combination target, were used to concentrate similar to the second session. The only difference between this task and baseline focus session was that the duration of target fixation was changed from 2 to 3 minutes in this task. The order of CST and CTCT tasks was randomized across participants. They were required to fixate on the 10 rings of the shooting target or on the bull’s eye of the combination target as best as possible.

### Task 4_2: Concentration with visual distractors (CVD)

While the participants were fixating their gaze on the center of a shooting target, a distractor (concentric circle with the same size as the 10 rings and inner 10 rings of the shooting target) appeared at 24 different positions of 3 distances (2, 4, 6 degrees of angular distance)^37^ and 8 directions (0, 45, 90, 135, 180, 225, 270, 315 degrees) around the shooting target in random order for 3–5 seconds (Table 2). The distractor appeared at each position for 3 times, so there were 72 trails in total. The duration of this task was approximately 6 minutes. Participants were requested to focus their eyes within the 10 rings of the shooting target and ignore the distractors.

### Task 4_3: Concentration with music distractors (CMD)

In this task, two different types of music were played in random order as auditory distractors when participants fixated on the shooting target. The first one is POP music distractor (CMD_pop_). A music containing lyrics (in Japanese) and named *Want to say love you loudly* from BAAD (theme song of Slam Dunk) was selected as the pop music distractor, which was generally used in emotional and exciting sports. Although music selected contained some lyrics pieces which might affect cognitive performance differently^38^, all the participants were under the same situation. Moreover, Chinese participants in our experiments did not understand the Japanese sentences. The first 3 minutes was played, and the audio intensity could reach 60–80 decibels. The second one is piano music distractor (CMD_Piano_). The piano music without lyrics titled *River flows in you* from Yiruma was selected. It was more calming and soothing than the pop music and the audio intensity reached around 50–60 decibels. Only the first 3 minutes was played. We designed these two types of music to find their different impact on the concentration of two groups so to find which music is better to focus the attention or to be benefit for the competition of athletes. Participants had heard both the pop and the piano music before.

### Session 5: Resting states

The CCT experiment ended with two resting state tasks which were the same as the first session. Participants were told to close their eyes without making any additional thoughts for 2 minutes in EC resting state and to fixate at the “ + ” shown on the center of the screen for 2 minutes in EO resting state. There was a short break between the two states. The end of the EC and EO states should be calculated offline using the 2 minutes interval. The second “S1-S2” trigger pair marks this resting state session.

## Data Records

### EEG data records

EEG data were acquired using a Neuroscan SynAmps2 EEG system and the Curry 8.0 recording software with 1000 Hz sampling rate and 24-bit A/D resolution (Neuroscan, Australia). The headbox of SynAmps2 consists of 64 unipolar EEG and 2 bipolar electrooculogram (EOG) channel. The ground electrode was placed on midway between Fz and Fpz. The reference electrode was Cz. All channels were located according to the extended 10–20 system. Conductive gel was used to reduce the impedance at each electrode. The impedance of most electrodes was kept below 10 kΩ, few might reach 20 kΩ (especially in the occipital lobe).

### ET data records

Eye movement data was simultaneously collected by an infrared video-based eye tracker (EyeLink Portable Duo, SR Research, Canada) at a sampling rate of 2000 Hz and an instrumental spatial resolution of 0.01 degree during all of the EEG paradigms. It was based on the traditional pupil and corneal reflection (CR) principle, where gaze locations on a calibration plane (usually a screen) are inferred from pupil-CR vectors through polynomial mapping^39^. Thirteen-point calibration was performed before the ABT and CCT experiments. In the validation step, the calibration was accepted only when the average gaze deviation of all points for single/both eyes was less than 0.5 degree. If one of the two eyes had a deviation larger than 0.5 degree, we would redo the calibration. If the number of repetitions was more than 3, participants would be asked to take a short break and performed the calibration again. If the number of the resting break was more than 2, we would just record the data of the eye reaching the calibration criterion. Before the ABT experiment, the average 13-point calibration of all participants left eyes was 0.314 ± 0.107 degree and that of their right eyes was 0.323 ± 0.136 degree. At the beginning of the CCT experiment, the average 13-point calibration of left eyes was 0.279 ± 0.104 degree and that of the right eyes was 0.282 ± 0.095 degree. Given the importance of the distance between fixation location and center of the screen in the CCT experiment, we calculated one central point calibration of all participants before CCT experiment. The central point calibration result of left eyes was 0.249 ± 0.147 degree and that of right eyes was 0.267 ± 0.143 degree. One central point drift calibration was performed prior to each task and we would recalibrate the eye-tracker if the error was larger than 1 degree.

### Data folders

The datasets^40^ were organized and described according to the Brain Imaging Data Structure (BIDS)^41^ for sharing and reusing data easily (Fig. 4). We began creating the dataset structure by creating three subfolders. The first and second subfolders were compressed into two zip format files and saved in the deposited dataset. Before the first subfolder, there was an excel file including all the participants demographic information and sport training information. For all the athletes, three types of training information were added in the columns, which were professional level, years of training and training load. The first subfolder included the results of two behavior tasks (ABT and SGT). The results of three questionnaires were in the second subfolder. Inside the third subfolder there were the raw ET and EEG data of all the athletes and controls. Each of 31athletes named ‘Athlete_Sub301’, ‘Athlete_Sub502’, etc. and each of 43 controls named ‘Control_Sub001’, ‘Control_Sub701’, etc. were created in the third subfolder and compressed into 74 zip format files. After each of the participant subfolders was decompressed, there were two experiments subfolders (ABT and CCT). In each experiment subfolder of the participant the raw EEG files (*.cdt) and ET file (*.edf) were stored separately. This operation had to be repeated for all the participants.

## Technical Validation

### Quality of ET data

According to the previous study^42^, three methods were chosen to examine the quality of ET data: proportion of valid data, inter-sample distance, and the distance of fixation to screen center. They measured different aspects of eye tracking data quality. The proportion of valid data measured whether the eyes could be detected by the eye-tracker, and whether the participants closed their eyes for extended periods during the experiment. The inter-sample distance measured whether the tracking was reliable when the eyes were detected by the eye-tracker. If the pupil or corneal reflection point could not be detected reliably, the data would have high frequency noise and the inter-sample distance would be high. The distance of fixation to screen center measured how accurate the tracking was and whether the participants were fixating at the screen center (which was required in most of the tasks). A large distance indicated that either the calibration was problematic, or the participants did not properly follow experiment instructions.

**Fig. 5:**
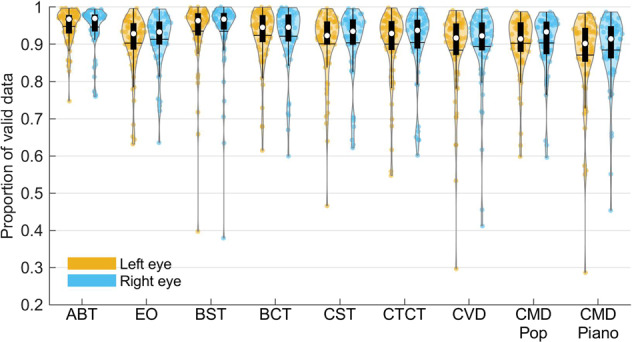
The distribution of proportion of valid ET data in each task. The colored dots in each violin plot represent data from each eye of the participants. The black horizontal line represents mean value. The white circle represents the median value. The black box represents the first and third quartile (Q1 and Q3). The black vertical line represents Q1–1.5 × IQR (interquartile range) and Q3 + 1.5 × IQR. ABT = Alertness Behavior Task; EO = Eye Open; BST = Baseline Shooting Target fixation; BCT = Baseline Combination Target fixation; CST = Concentration Shooting Target; CTCT = Combination Target Concentration Task; CVD = Concentration with Visual Distractors; CMD Pop = Concentration with Music Distractors (pop music); CMD Piano = Concentration with Music Distractors (piano music).

We first detected periods of invalid data (due to blinks or tracking loss) by an eye-movement event detection algorithm with adaptive threshold^43^. This algorithm also marked the gaze data transients immediately before and after the blinks as invalid data as well. The rest of the data were considered as valid regardless of the noise level. The noise remaining in the data was primarily high frequency noise due to unreliable tracking, and jerks in gaze data probably  due to partial blinks (blinks without the eyelids being completely closed, so the pupil could still be tracked but its center were deviated). The examples of the two types were given in the supplementary information. The proportion of valid data in a task was the ratio between the number of valid data samples and the total number of data samples in that task. Data from each eye were processed separately. As shown in Fig. 5, the average proportion of valid data of all tasks and sessions were around 0.90 (0.87 – 0.95), but a small number of data samples had a much lower proportion. These datasets either had a significant tracking loss (SFig. 1b), or had a high blink rate (SFig. 1c). An example data with high proportion valid value was shown in supplementary figure (SFig. 1a) for comparison. In general, good ET data should have low data loss rate, low noise level, and show that the participant was actively following task instructions. In the supplementary information we provided some examples of how data of different quality look like.

**Fig. 6:**
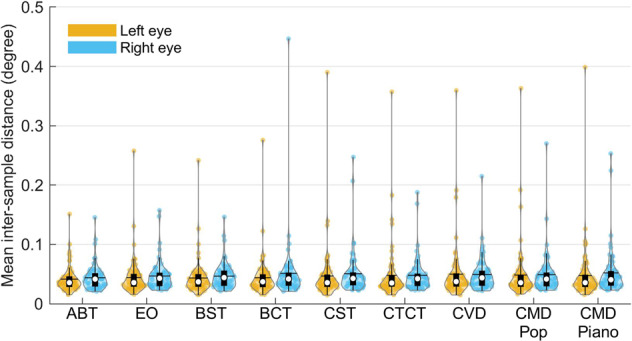
The distribution of average inter-sample distance of gaze position in each task. The colored dots in each violin plot represent data from each eye of the participants. The black horizontal line represents average value. The white circle represents the median value. The black box represents the first and third quartile (Q1 and Q3). The black vertical line represents Q1–1.5 × IQR (interquartile range) and Q3 + 1.5 × IQR. ABT = Alertness Behavior Task; EO = Eye Open; BST = Baseline Shooting Target fixation; BCT = Baseline Combination Target fixation; CST = Concentration Shooting Target; CTCT = Combination Target Concentration Task; CVD = Concentration with Visual Distractors; CMD Pop = Concentration with Music Distractors (pop music); CMD Piano = Concentration with Music Distractors (piano music).

To quantify the noise level of the ET data, we then calculated the angular distance between consecutive gaze samples in each of the valid data periods. The values of inter-sample distance were calculated for each eye separately. The mean inter-sample distance of most participants fell below 0.05 degrees, but some participants’ data had much higher values (Fig. 6). For clearer comparison, SFig. 2 showed example data of low (0.015 degree) and high (0.195 degree) inter-sample distance of gaze position in the supplementary file. The data with high inter-sample distance is noticeably noisier.

**Fig. 7:**
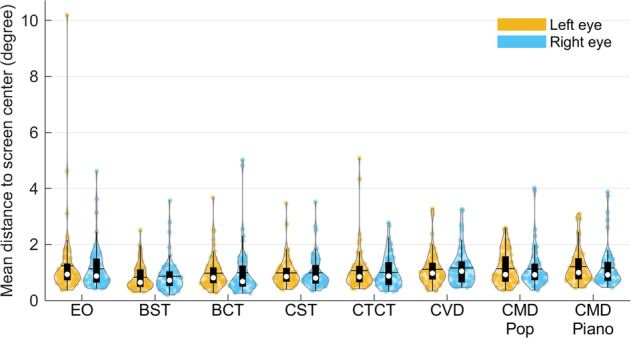
The distributions of fixation distance to screen center during the CCT sessions. The colored dots in each violin plot represent data from each eye of the participants. The black horizontal line represents average value. The white circle represents the median value. The black box represents the first and third quartile (Q1 and Q3). The black vertical line represents Q1–1.5 × IQR (interquartile range) and Q3 + 1.5 × IQR. EO = Eye Open; BST = Baseline Shooting Target fixation; BCT = Baseline Combination Target fixation; CST = Concentration Shooting Target; CTCT = Combination Target Concentration Task; CVD = Concentration with Visual Distractors; CMD Pop = Concentration with Music Distractors (pop music); CMD Piano = Concentration with Music Distractors (piano music).

Finally, we calculated the angular distance of all valid gaze data to the screen center in the tasks of CCT experiment. The mean disparity across participants lay between 0.85 to 1.26 degrees (Fig. 7). It was larger than the initial calibration 0.5 degree, which might be caused by failure to keep fixation at the target due to lose concentration. However, other reasons, such as accumulated head motion during the experiment, different radius of target or calibration error, should be taken into account especially in setting the threshold degree of losing visual attention when some participants’ data had much larger disparity. Notably, the disparity of one participant was larger than 10 degrees (Fig. 7), and this was caused by very unstable recognition of the eye during the second resting state session. In the supplementary file SFig. 3 showed examples of low and high disparity in the rest of the data. One can see that the participant in SFig. 3b frequently directed fixations away from the screen center, and suggests that the participant was not fully concentrated on the experiment.

We also found that some participants made partial blinks, which cannot be well characterized by the above three methods. This happened when the participant blinked without fully closing the eyelid, so the pupil could still be detected but was severely distorted. Partial blinks generate short spikes in the gaze data without data loss (SFig. 4), so they can’t be recognized by the blink detection algorithm provided by device manufacturer. We advise users to specifically take care of this event when processing the eye-tracking data.

### ET data validation

**Fig. 8:**
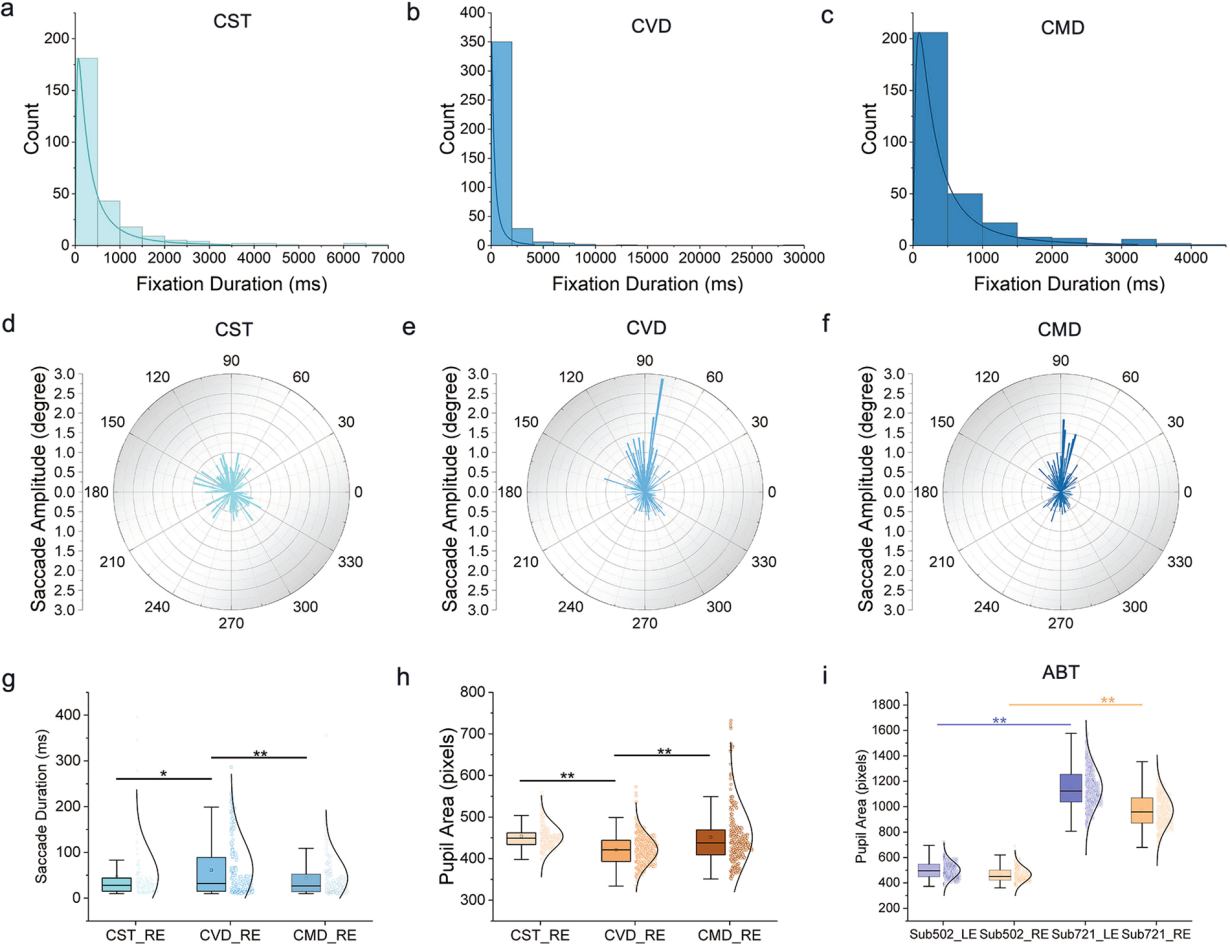
Comparisons of eye movement properties between different sessions or participants. (**a**–**c**) The distributions of right-eye fixation duration of Sub502 in the CST (**a**), CVD (**b**) and CMD (**c**) tasks, respectively. Curve lines were lognormal distributions. (**d**–**f**) Polar histogram of right-eye saccade amplitude of Sub502 in the CST (**d**), CVD (e) and CMD (**f**) tasks respectively. (**g**) For the athlete Sub502, the right-eye (RE) saccade duration in the CVD was significantly longer than that in the CST and CMD. (**h**) The right-eye pupil area of Sub502 in the CVD was significantly smaller than that in the CST and CMD. (**i**) The pupil area of the athlete Sub502 was significantly smaller than that of the control Sub721 both in left-eye (LE) and right-eye during the ABT experiment. (*p < 0.05; **p < 0.01) CST = Concentration Shooting Target; CVD = Concentration with Visual Distractors; CMD = Concentration with Music Distractors.

Both ET data and EEG data of all participants can be deeply analyzed for various purposes. As shown above, eighteen tasks of five sessions were in the CCT experiment. Each task had different initial research aims. Here, the ET data of one modern pentathlon athlete (Sub502) and one college student (Sub721) in the dataset were randomly selected for analysis as an example. Saccades, fixations and blinks were detected by the built-in algorithm of the EyeLink tracker^44^. Saccades with amplitude less than 0.2 degree or duration less than 10 ms were removed^43^. Taking the athlete’s right-eye data under different concentration tasks as an example, the results showed that the single fixation duration was longer and number of fixation event was less (Fig. 8a–c), the amplitude of saccade was smaller (Fig. 8d–f), the duration of saccade was shorter (Fig. 8g) and the pupil area was larger (Fig. 8h) in CST task compared to the CVD and CMD tasks. It suggested that the eye movement would show different characteristics in different visual or auditory distractor tasks.

In the same task, such as ABT experiment, the pupil area of left and right eye of the athlete were both significantly smaller than that of the control (Fig. 8i). The result was in line with our expectations and previous literatures that size-reduced pupil could enhance visual discrimination during the focus, the smaller the change of pupil area, the more stable the concentration state was and the less the energy cost^45,46^. The analysis above suggests that master athletes may have different eye movement characteristics from the control students. Many questions such as eye movement differences between groups or eye movement characteristics of concentration or eye movement biomarker of different sports etc. can be further studied by more researchers using this dataset.

### EEG data validation

**Fig. 9:**
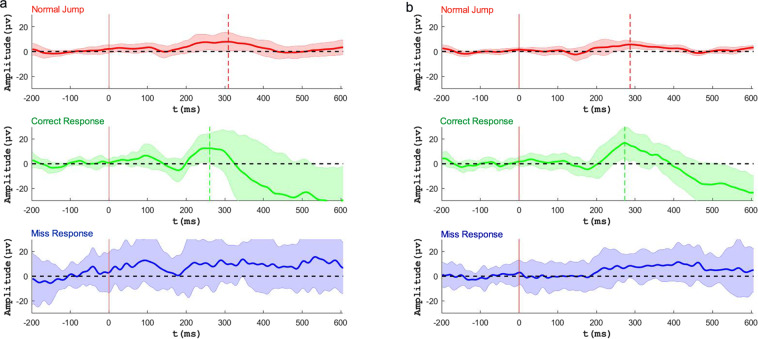
P300 wave of normal jump without response, correct response and miss response trials of (a) fifteen modern pentathlon athletes, and (b) fifteen control college students (Mean ± SD). Red solid vertical lines represent the starting point of the response. Red and green dotted vertical lines represent the time of peak P300 value of normal jump and correct response, respectively.

We used the P300 event-related potentials (ERP) as an index of attention state for the EEG data of ABT experiment^47–49^.In ABT tasks, elite athletes were expected to have less misses than controls due to the high level of attention. The filter used during EEG online recording was 0–500 Hz. Online filters were Bessel IIR filters with slope of 12 dB/oct. For the offline analysis, data of each participant were preprocessed in EEGLAB. They were bandpass filtered to a frequency range of 0.1–40 Hz by a zero-phase Hamming-windowed-sinc FIR filter. Artifacts like eye movements were removed by using an open-source plug-in AAR for EEGLAB^50^, which performed automatic electrooculogram (EOG) artifact correction using blind source separation (BSS) and identified the EOG components using fractal analysis. The source code is available on the website (https://github.com/germangh/eeglab_plugin_aar). Based on previous studies^51,52^, we selected 15 channels, from frontal, central and parietal brain areas, with which attention was invoked correlated significantly (F1, Fz, F2, Fc1, Fcz, Fc2, C1, Cz, C2, Cp1, Cpz, Cp3, P1, P3, P5). We compared the P300 wave of normal jump without response, correct response and miss trails. The data was epoched from the 200 ms before the white dot turned red to the 600 ms after. Each epoch was baseline-corrected by subtracting the mean voltage from the 200 ms prior to the onset. Fifteen athletes and fifteen control students were randomly selected for ERP analysis. P300 amplitudes were calculated by the mean of 15 ms before and after peak. Independent two-sample t-test was performed to assess the differences on P300 amplitude from two groups or in different conditions. The results of two groups were presented in Fig. 9.

Because ERP was the measurement of the brain’s psychological response to specific sensory, cognitive or motor events from the external environment. Among them, P300 was a kind of ERP, which was an endogenous and special evoked potential related to cognitive function. P300 could be induced by visual, auditory and somatosensory stimuli and used to identify neural activity associated with cognitive processes in the human brain^53^. Larger P300 amplitude were associated with superior performance in the cognitive functions such as attention^54^. Therefore, when participants made the correct response, it indicated that they had focused on capturing the target stimulus and the P300 wave would be observed. However, when they missed the double-jumped red dot, the mind of participants might be wandering and didn’t concentrate on the target. There were no some obvious mental activities such as decision-making and attention, so P300 would not be observed. As the Fig. 9a,b showed, P300 wave was produced by both groups when they responded correctly in normal jump and double jump conditions, and no distinct P300 was found when participants missed the double-jumped red dot. This suggested that whether athletes or controls were all attentional when they responded correctly to both target and non-target stimuli, and were non-attentional when they missed the target stimuli. In addition, this comparison showed significant evidence of high quality of the data. We also found that the amplitude of the P300 wave of the correct response for the target stimuli was significantly higher than that for the non-target stimuli (normal jump) (Target = 16.74 ± 10.71μv, Non-target = 8.3 ± 5.06μv; p < 0.01). In normal jump condition, the amplitude of P300 signals generated by athletes were significantly higher than those generated by controls (Athletes = 9.91 ± 6.05μv, Controls = 6.7 ± 3.31μv; p < 0.05). When they responded correctly in double jump condition, the P300 amplitude of athletes showed a little higher than that of controls but no significance between them (Athletes = 17.05 ± 10.79μv, Controls = 16.44 ± 10.61μv; p > 0.05). Meanwhile, the P300 latency of athletes was shorter than that of controls in correct responses (Athletes = 261 ms, Controls = 273 ms). These results were in line with the expectation that elite athletes might have higher level of concentration than controls.

Plenty of existing researches have manifested the close relation between EEG and attention state. By dividing EEG into multiple different rhythms and analyzing the power characteristics, the attention level can be evaluated^55–57^. For instance, an adaptive agent was developed to monitor student attention and recapture diminishing attention in real time using EEG^55^. Theta/beta ratio of EEG was taken as a potential biomarker for attentional control and the interaction between stress and attention was investigated^56^. Besides, a wireless and wearable EEG system was designed to evaluate driver attention level during driving tasks^57^. Therefore, we try to validate the effectiveness of the EEG dataset by establishing a mapping from the original signal to the objective attention state.

The signals of EC, BST and BCT sessions were analyzed and the attentional states were compared among the three different experiment sessions after the EEG data of each participant were Notch filtered to remove 50 Hz power line noise for acquiring better preprocessed EEG signal. Some previous researches suggested that the EEG alpha (8–13 Hz) and beta (14–20 Hz) band were closely related to the attention state and a lower alpha/beta ratio implied a potentially higher attention level^58,59^. Specifically, suppression of alpha rhythm was related to more concentrated task engagement^58^, and increased beta rhythm was related to high performance in sustaining attentional processes^59^. Therefore, we took the ratio of alpha and beta power as an index to compare the three selected sessions and expected the elite athletes had lower alpha/beta ratio in the tasks, which made sense that professional athletes were good at focusing their attention on the shooting targets.

**Fig. 10:**
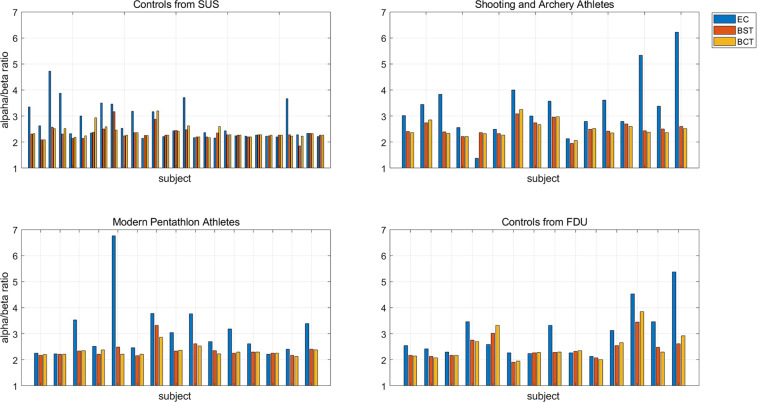
The mean alpha/beta band power ratio of all the participants during the eye-closed (EC) resting state, baseline shooting target (BST) fixation task, and baseline cross-circle combination target (BCT) fixation task.

Figure 10 Showed that the alpha/beta ratio of most participants were higher in the EC resting state than that in the BST or BCT task as expected, which was consistent with previous findings^58,59^. The ratios of different groups were compared in the Table 3. It seemed that shooting and archery athletes got higher ratio during each session. A possible explanation for this phenomenon was that the least attention engagement was required for the shooting and archery athletes in order to accomplish the same task. More researches need to be further explored by using this dataset.

**Table 3:** The ratio value of power spectrum of alpha band and beta band in different groups.

Group	EC	BST	BCT
SUS controls	2.7388	2.3299	2.3727
Shooting and Archery Athletes	3.3462	2.5210	2.5030
Modern pentathlon Athletes	3.1225	2.3731	2.3280
FDU controls	3.0022	2.4413	2.5024

## Usage Notes

### Task order

Focusing on shooting target or combination target was released randomly in the baseline focus session and concentration without distractors task. In the intervention task session, MT or SGT was trained randomly and the detail order of each subject was listed in their demographic information.

### Blank ET data

ET data was blank during EC resting state and MT intervention because the eyes were closed. ET data was not recorded during the SGT intervention because the stimulus was displayed on an iPad instead of the stimulus screen. In the dataset, the ET data of Sub704 was blank for not recording triggers correctly.

### Definitions of terms

In the dataset there were two independent experiments which might include some tasks classified into different sessions. We used three terms to illustrate the experimental procedures. To avoid misunderstanding we clarified them here. ‘*Experiment*’, a set of activities performed by the participant in each experiment. The simultaneous EEG and ET data were continuously recorded in each experiment. That is, the data of one experiment included both the data of activities and the data of break resting between two activities. ‘*Session*’, the activities could be classified into some sessions by the purpose of research in each experiment. ‘*Task*’, a task was one activity during the simultaneous EEG and ET data acquisition. Each task has a start trigger and an end trigger or duration (e.g., resting state).

## Supplementary information

Supplementary Information

## Data Availability

The Matlab data variables can be obtained by exporting the EEG raw files (*.cdt) and ET raw files (*.edf) using MATLAB R2021a (MathWorks, Natick, MA, USA) and EEGLAB toolbox (http://sccn.ucsd.edu/eeglab/). The codes for analyzing the ET and EEG data were compressed into a zip format file and shared with the dataset.
