# Supplementary material for: A simultaneous electroencephalography and eye-tracking dataset in elite athletes during alertness and concentration tasks
Source: Sci Data. 2022 Aug 2;9:465. doi: 10.1038/s41597-022-01575-0 (PMC9345900; doi:10.1038/s41597-022-01575-0)
Supplement: Supplementary file 1 — Supplementary Information [file 41597_2022_1575_MOESM1_ESM.pdf]

# Supplementary Information


| Order Number | Contents | Page |
|---|---|---|
| 1 | **The explanation of ET data validation** | 1/6 |
| | SFig.1 Examples of data in the alertness task with high and low valid data proportion | 2/6 |
| | SFig.2 Examples of low and high inter-sample distance of data in the alertness task | 3/6 |
| | SFig.3 Examples of low and high distance to screen center in eye-open resting state data | 4/6 |
| 2 | **The examples of the noise that was left in the data** | 5/6 |
| | SFig.4 Example of partial blinks in eye-tracking data | 5/6 |
| 3 | **EEG acquisition process** | 5/6 |
| 4 | **Shooting Target Size** | 6/6 |
| | STable 1. Shooting target size in the Olympic Games and on the screen | 6/6 |


## 1 The explanation of ET data validation

In general, good data should have low data loss rate, low noise level, and show that the participant was actively following task instructions.

Here we give some examples of how data of different quality look like. The participant index, task name and value of data quality measures are shown in the title of figures. Note that the data quality values displayed in figure titles are calculated from the entire data of the participant in this task, not from the displayed period. In the Alertness (ABT) task the participants' eyes were usually tracking the move dot on the circle, so the gaze data had a sinusoidal shape. In the rest of tasks, the participants' eyes should always fixate at the screen center.

(1) Proportion of valid data (denoted as p(valid)) (SFig.1)

(2) Inter-sample distance (SFig.2)

The gaze data in SFig.2b looked "thicker" compared to the data in SFig.2a due to the presence of high-frequency noise. This suggested that tracking of the pupil or corneal reflection point was noisy during this period of the experiment. Note that blinks were excluded when calculating inter-sample distance.

(3) Distance to screen center (SFig.3)

The participant in SFig.3a was fixating very accurately at the screen center. The participant in SFig.3b fixated away from the fixation point at the screen center for four times (around 177-187, 193-197, 205-206, and 210-217 seconds) in the figure. This suggested that the participant was not fully concentrated on the experiment during this period.

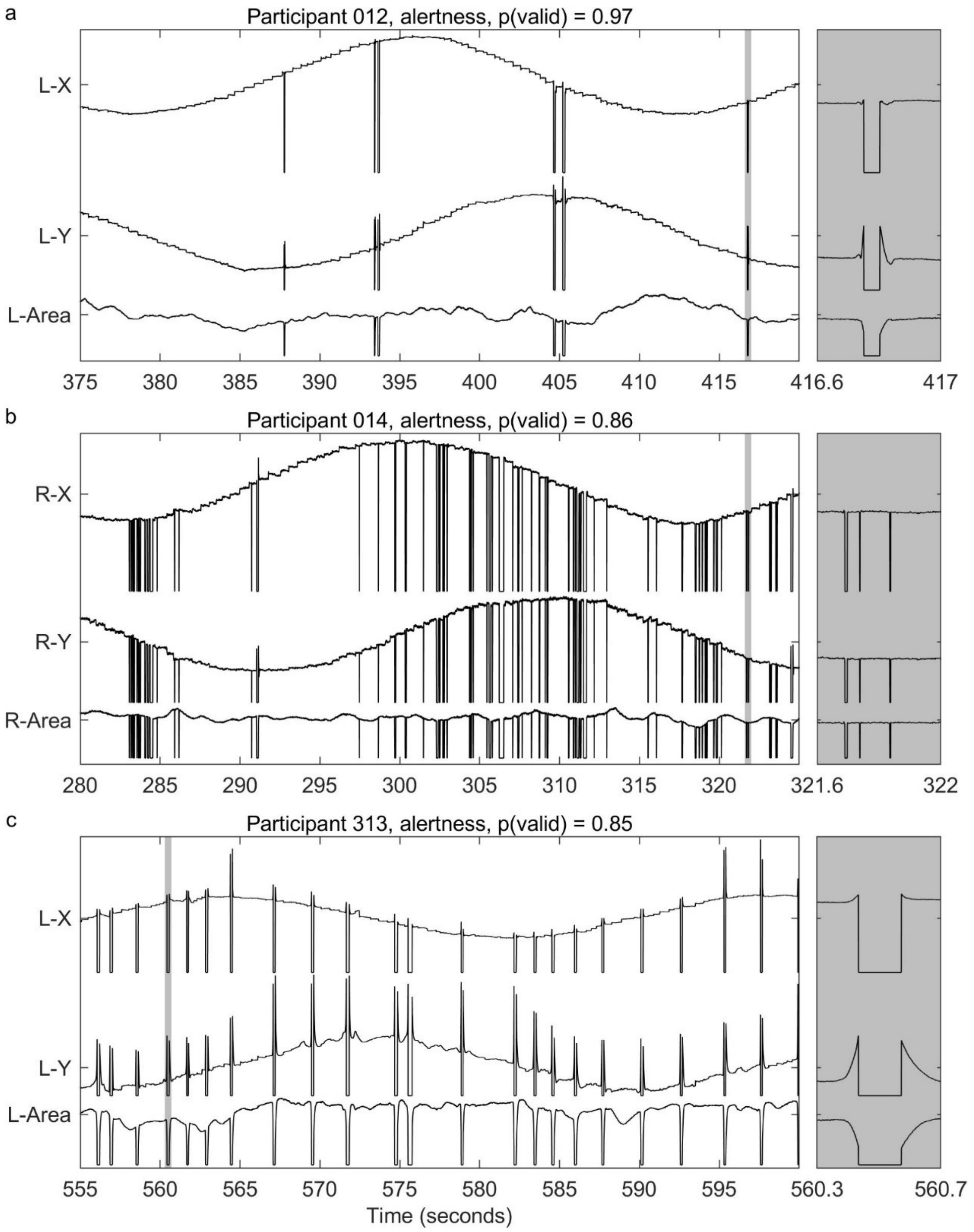


**SFig. 1** Examples of data in the alertness task with (a): high valid data proportion; (b): low valid data proportion due to unstable tracking; and (c): low valid data proportion due to high blink rate. The three grey axes on the right are enlarged views of the grey periods in the left axes. Note how data loss due to unstable tracking in (b) looks different than blinks in (a) and (c). The mean proportion valid values displayed in figure titles are calculated from the entire data of the participant in this task, not from the displayed period. L/R-X: left/right horizontal gaze position; L/R-Y: left/right vertical gaze position; L/R-Area: pupil area of the left/right eye.

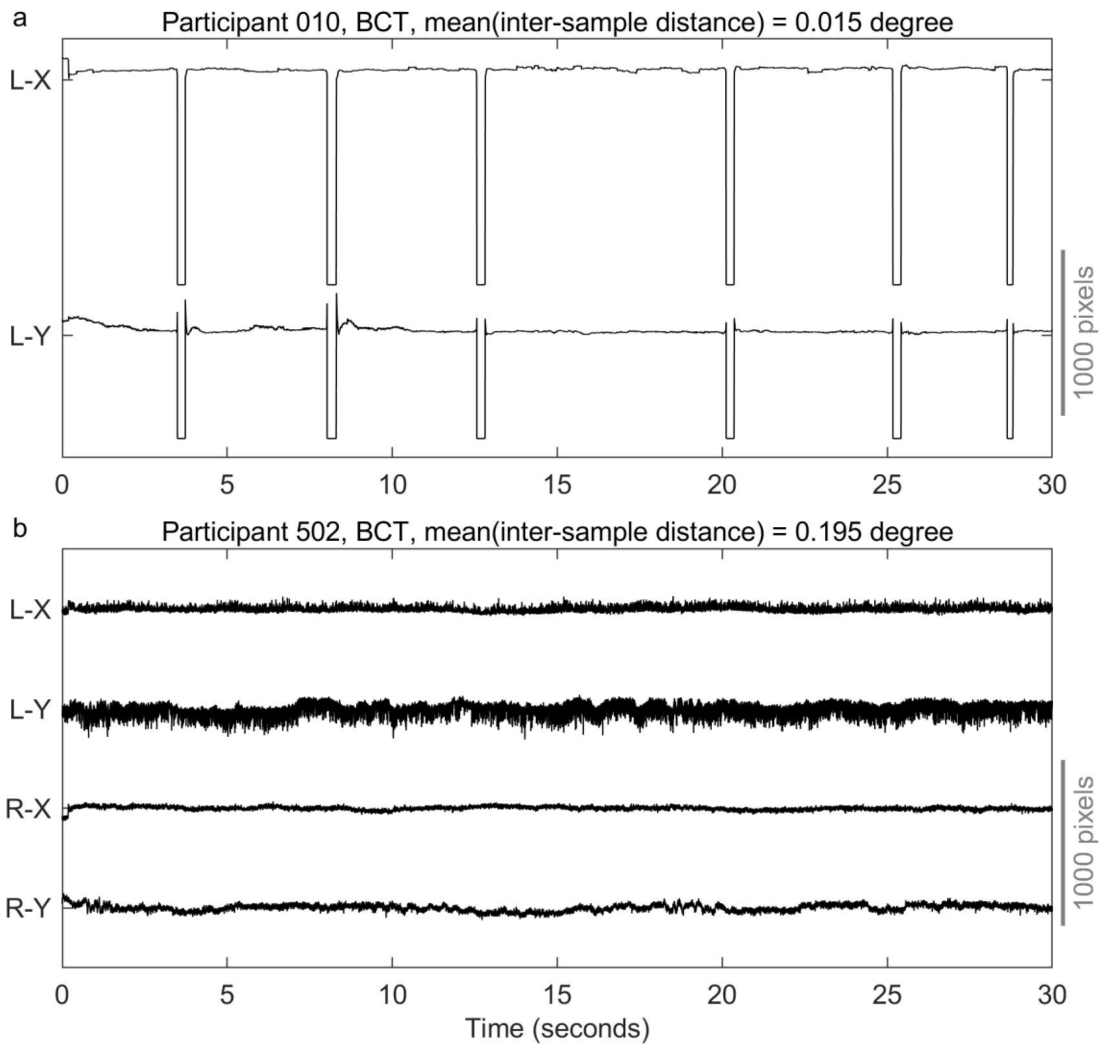


**SFig. 2** Examples of low (a) and high (b) inter-sample distance of data in the alertness task. The limits and scales of the y-axis of the two panels are the same. The inter-sample distance was calculated only in valid data (blinks were excluded). If binocular data were presented it was first calculated for each eye and then averaged together. Note that the value of mean inter-sample distance in figure titles are calculated from the entire data of the participant in this task, not from the displayed period. L/R-X: left/right horizontal gaze position; L/R-Y: left/right vertical gaze position.

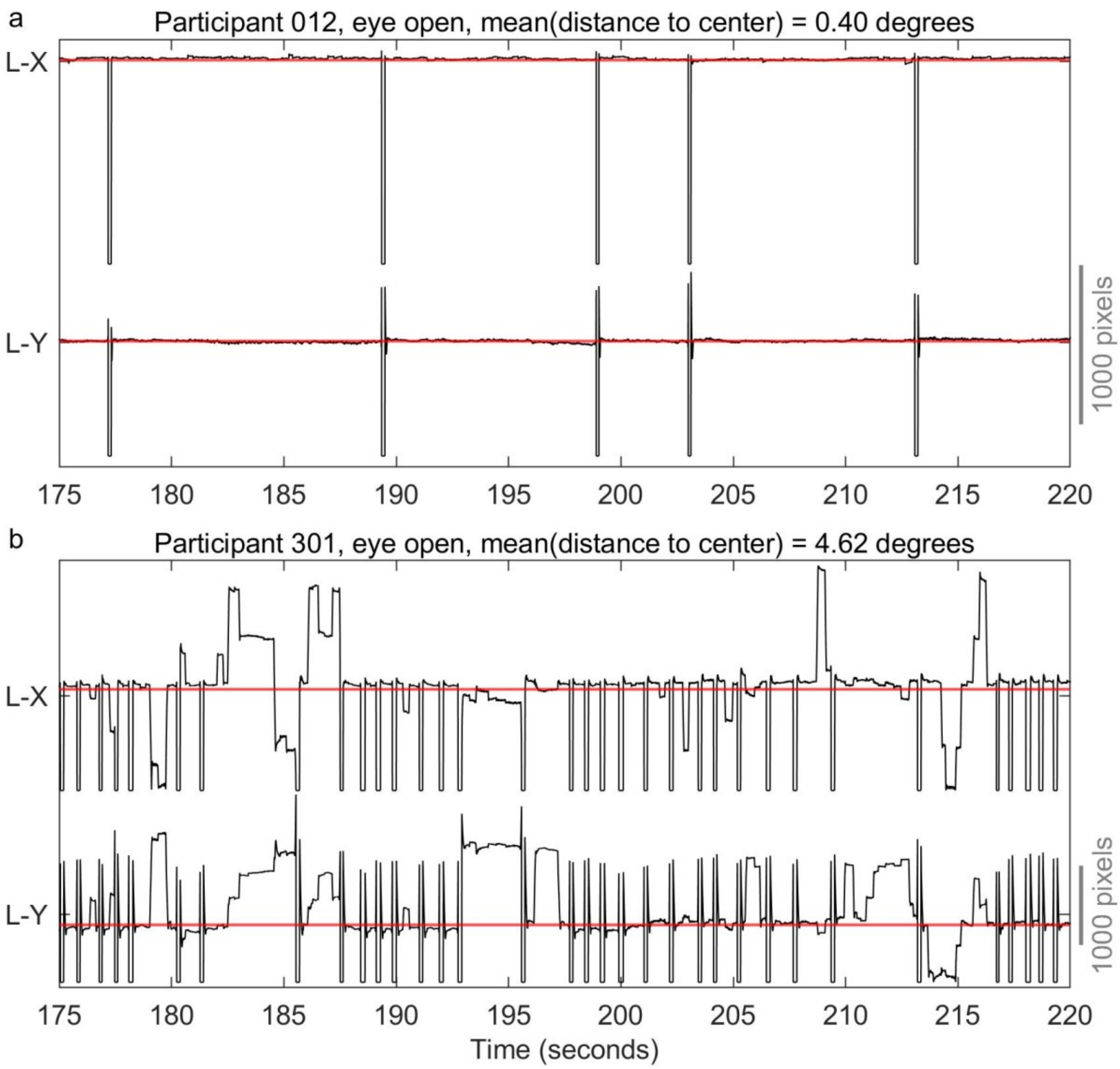


**SFig. 3** Examples of low (a) and high (b) distance to screen center in eye-open resting state data. The red lines represent the horizontal and vertical position of the screen center (1280 and 720 pixels, respectively) that the participant should fixate. The black lines are the recorded gaze data. The distance to screen center was calculated only in valid data (blinks were excluded). Note that the value of mean distance to center in figure titles are calculated from the entire data of the participant in this task, not from the displayed period. L-X: left horizontal gaze position; L-Y: left vertical gaze position.

## 2 The examples of the noise that was left in the data

The noise remaining in the data was primarily high frequency noise due to unreliable tracking, and jerks in gaze data probability due to partial blinks (blinks without the eyelids being completely closed, so the pupil could still be tracked but its center were deviated). The example of high-frequency noise was provided in SFig.2b and the example of partial blinks was provided in SFig.4.

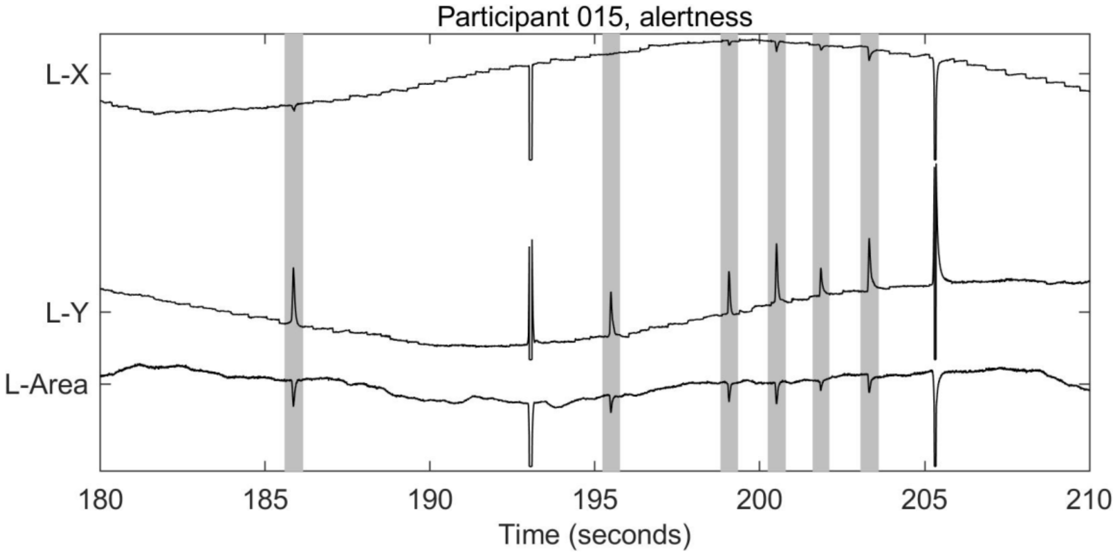


**SFig.4** Example of partial blinks in eye-tracking data, marked in gray regions. L-X: left horizontal gaze position; L-Y: left vertical position; L-AREA: pupil area of the left eye.

## 3 EEG acquisition process

The EEG acquisition process had 4 steps.

(1). Equipment debugging. Before placing the EEG cap, we would switch on the equipment and debug the instrument to ensure smooth recording.

(2). Preparation before collection, including informing the subjects of hair washing in advance, the purpose of EEG collection and the non-harm of the collection process to relieve the tension of the subjects.

(3). Place EEG cap and connect VEOU/VEOL (vertical eyeball, left eye), HEOR/HEOL (horizontal eyeball) and bilateral mastoid process (M1/M2) electrode in accordance with international standards. Wipe scalp with an appropriate amount of conductive cream or abrasive cream to reduce the impedance to less than 10 kΩ as far as possible, so as to reduce the influence of oil and cutin.

(4). Event markers in the recording process were used to mark the electrode dropping and the state of the subject in the recording process for subsequent processing.

## 4 Shooting Target Size

STable 1. Shooting target size in the Olympic Games and on the screen

| Ring | Diameter in Event (mm) | Degree on Screen (°) | Color |
|---|---|---|---|
| 1 | 155.5 | 0.8909 | white |
| 2 | 139.5 | 0.7992 | white |
| 3 | 123.5 | 0.7076 | white |
| 4 | 107.5 | 0.6159 | white |
| 5 | 91.5 | 0.5242 | white |
| 6 | 75.5 | 0.4326 | white |
| 7 | 59.5 | 0.3409 | white |
| 8 | 43.5 | 0.2492 | black |
| 9 | 27.5 | 0.1576 | black |
| 10 | 11.5 | 0.0659 | black |
| Inner 10 | 5 | 0.0286 | black |
